# Early selexipag initiation and long-term outcomes: insights from randomised controlled trials in pulmonary arterial hypertension

**DOI:** 10.1183/23120541.00456-2022

**Published:** 2023-01-16

**Authors:** J. Gerry Coghlan, Sean Gaine, Richard Channick, Kelly M. Chin, Camille du Roure, J. Simon R. Gibbs, Marius M. Hoeper, Irene M. Lang, Stephen C. Mathai, Vallerie V. McLaughlin, Lada Mitchell, Gérald Simonneau, Olivier Sitbon, Victor F. Tapson, Nazzareno Galiè

**Affiliations:** 1 Royal Free Hospital, London, UK; 2 Mater Misericordiae University Hospital, Dublin, Ireland; 3 David Geffen School of Medicine, University of California Los Angeles, Los Angeles, CA, USA; 4 UT Southwestern Medical Center, Dallas, TX, USA; 5 Actelion Pharmaceuticals Ltd, a Janssen Pharmaceutical Company of Johnson & Johnson, Global Medical Affairs, Allschwil, Switzerland; 6 National Heart and Lung Institute, Imperial College London, London, UK; 7 Department of Respiratory Medicine, Hannover Medical School and German Center for Lung Research, Hannover, Germany; 8 Division of Cardiology, Medical University of Vienna, Vienna, Austria; 9 Johns Hopkins University School of Medicine, Baltimore, MD, USA; 10 University of Michigan, Ann Arbor, MI, USA; 11 Actelion Pharmaceuticals Ltd, a Janssen Pharmaceutical Company of Johnson & Johnson, Statistics & Decision Sciences - Medical Affairs and Established Products, Allschwil, Switzerland; 12 Hôpital Bicêtre, Université Paris-Sud, Le Kremlin Bicêtre, France; 13 Cedars-Sinai Medical Center, Los Angeles, CA, USA; 14 Alma Mater Studiorum, University of Bologna and IRCCS-S.Orsola University Hospital, Bologna, Italy

## Abstract

Further understanding of when to initiate therapies in pulmonary arterial hypertension (PAH) is important to improve long-term outcomes. *Post hoc* analyses of GRIPHON (NCT01106014) and exploratory analyses of TRITON (NCT02558231) suggested benefit of early selexipag initiation on long-term outcomes, despite no additional benefit *versus* initial double combination on haemodynamic and functional parameters in TRITON. *Post hoc* analyses investigated the effect of early selexipag initiation on disease progression and survival in a large, pooled PAH cohort. Data from newly diagnosed (≤6 months) PAH patients from GRIPHON and TRITON were pooled. Patients on active therapy with selexipag (pooled selexipag group) were compared with those on control therapy with placebo (pooled control group). Disease progression end-points were defined as per the individual studies. Hazard ratios (HR) and 95% CI for time to first disease progression event up to end of double-blind treatment (selexipag/placebo) +7 days and time to all-cause death up to end of study were estimated using Cox regression models. The pooled dataset comprised 649 patients, with 44% on double background therapy. Selexipag reduced the risk of disease progression by 52% *versus* control (HR: 0.48; 95% CI: 0.35–0.66). HR for risk of all-cause death was 0.70 (95% CI: 0.46–1.10) for the pooled selexipag *versus* control group. Sensitivity analyses accounting for the impact of PAH background therapy showed consistent results, confirming the appropriateness of data pooling. These *post hoc*, pooled analyses build on previous insights, further supporting selexipag use within 6 months of diagnosis, including as part of triple therapy, to delay disease progression.

## Abstract 2

**This *post hoc* pooled analysis of GRIPHON and TRITON patients with a diagnosis of ≤6 months suggests that early targeting of the prostacyclin pathway with selexipag may be beneficial in delaying disease progression in a broad PAH population** https://bit.ly/3CocEBe

## Introduction

Pulmonary arterial hypertension (PAH) is a rare, progressive disorder [1, 2]. Several pathogenic pathways contribute to its progression, including the prostacyclin, endothelin and nitric oxide pathways, which can be targeted by medical treatment [1–5]. In clinical practice, drugs targeting the prostacyclin pathway, including the oral prostacyclin receptor (IP receptor) agonist selexipag, are typically initiated in PAH patients years after diagnosis [6, 7]. Selexipag is indicated to delay progression of PAH, including a reduced risk of hospitalisation. Recent analyses suggest that these effects might be optimised by early initiation of selexipag [8–10].

The TRITON randomised controlled trial assessed the impact of early use of selexipag as triple oral therapy (macitentan, tadalafil, selexipag) *versus* double oral therapy (macitentan, tadalafil, placebo) in newly diagnosed, treatment-naive patients with PAH, with a primary end-point of change in pulmonary vascular resistance at week 26 [8]. Though both treatment groups showed clinically meaningful improvements, no differences were observed between the treatment groups for either the primary end-point or for the secondary end-points of 6-min walk distance (6MWD) and World Health Organization functional class (WHO FC) assessed over a short-term period of 26 weeks. TRITON also evaluated PAH disease progression and survival in a blinded manner over the long-term (median follow-up duration was 77.6 and 75.8 weeks in the initial triple and initial double therapy groups, respectively). Assessment of these end-points suggested a potential benefit with initial triple *versus* initial double combination therapy. In the exploratory analysis of the secondary end-point of time to disease progression, the hazard ratio (HR) for initial triple *versus* initial double therapy for occurrence of an event was 0.59 (95% confidence intervals (Cl): 0.32–1.09) [8]. Observing an improvement in long-term outcomes without an effect on short-term parameters has been made in other disease areas but not in PAH [8]. It is therefore important to take the opportunity to further explore the potential for long-term benefits.

These findings regarding long-term outcomes in the newly diagnosed TRITON population were consistent with observations in the predominantly prevalent GRIPHON population (n=1156) [11]. In GRIPHON, selexipag reduced the risk of disease progression by 40% (composite primary end-point), irrespective of whether patients were receiving background PAH therapy. The effect of selexipag on 6MWD and WHO FC was more modest, particularly in patients receiving background therapy [11]. In a recent *post hoc* analysis from GRIPHON, selexipag treatment within 6 months of diagnosis reduced the risk of disease progression by 55% compared with placebo, including in a small proportion of patients on PAH background therapy, providing evidence supporting the benefits of early treatment with selexipag to delay the progression of PAH [9].

This analysis further investigated the signal observed in TRITON for improved long-term outcomes by pooling PAH patients from the GRIPHON and TRITON clinical trials and assessing the impact of initiating selexipag within 6 months of diagnosis on disease progression and survival.

## Methods

The data sharing policy of Janssen Pharmaceutical Companies of Johnson & Johnson is available at https://www.janssen.com/clinical-trials/transparency. As noted on this site, requests for access to the study data can be submitted through Yale Open Data Access (YODA) Project site at http://yoda.yale.edu.

### Study design

For this *post hoc* analysis, data from the GRIPHON (NCT01106014) and TRITON (NCT02558231) studies were pooled together, based on similarities between the two studies in rationale, objectives and study design (*e.g.*, study treatments, observation time, end-points, adjudication of events). An overview of the individual study designs is shown in supplementary table S1. Censoring rules and follow-up between the two studies were aligned following previously described methodology [12].

### Patient selection

This *post hoc* analysis included newly diagnosed patients from GRIPHON and TRITON, *i.e.*, those with a diagnosis of PAH within the previous 6 months. The individual patient selection criteria by study are shown in supplementary table S1. Written informed consent was obtained for all patients before participation. Both studies adhered to the principles outlined in the amended Declaration of Helsinki and the study protocols were approved by the local institutional review board or independent ethics committee at each study site. Patients randomised to selexipag in each study were pooled to form the pooled selexipag group, and patients randomised to placebo in each study were pooled to form the pooled control group.

### Analyses objectives and end-points

The main objective of this analysis was to investigate the effect of early initiation of selexipag on long-term outcomes. For the analysis of disease progression up to end of treatment period, the time to first disease progression event end-point was defined for patients with ≥1 event as the time from randomisation until the first event or, for patients with no event, until end of treatment +7 days in GRIPHON or until end of treatment +7 days or end of main observation period +7 days in TRITON (whichever was earliest). The individual components of each disease progression end-point for each study can be found in supplementary table S2. Other end-points were time to all-cause death (defined as time from randomisation to all-cause death up to study closure in GRIPHON or TRITON) and safety (treatment emergent adverse events (AEs), serious AEs and AEs leading to study treatment discontinuation).

### Statistical analyses

The treatment effect of selexipag on time to disease progression up to end of treatment period or time to all-cause death up to end of study was estimated with HR and corresponding 95% CI. For the main analysis of time to disease progression or time to all-cause death, Cox proportional hazard regression was performed, using a model which included treatment, age, sex, race, PAH aetiology, region, WHO FC, 6MWD, N-terminal pro-brain natriuretic peptide (NT-proBNP) levels and study as covariates. Covariates were chosen based on their clinical importance in PAH. The Kaplan–Meier (KM) method was used to estimate event-free rates and their 95% CIs. KM curves in graphical presentations were truncated when <10% of patients remained, as per Pocock's Rule [13], and CIs were constructed using Greenwood's formula [14]. To explore potential differences between studies in the time to disease progression end-point, the censoring rules pattern and median follow-up for each study was summarised using reverse KM methodology [12, 15].

To explore the time-varying intervention effect, an additional sensitivity analysis was performed using the same model as the main analysis but adding the variable of concomitant (post-baseline) use of endothelin receptor antagonist (ERA) and phosphodiesterase type 5 inhibitor (PDE-5i) as a time-dependent covariate [16]. Owing to the relatively small number of events and patients, a subgroup analysis was performed in patients receiving an ERA and PDE-5i at baseline using a model that included only treatment, region, WHO FC and study as covariates. As this was not a randomised controlled trial and in order to explore potential differences and confounding factors between the pooled control and pooled selexipag groups, sensitivity analyses for time to disease progression using simple exact matching and propensity score weighting were performed (supplementary methods 1). Pooled safety data, collected until end of treatment +30 days for TRITON or end of study treatment +7 days for GRIPHON, were analysed descriptively as categorical variables.

## Results

### Patient disposition and characteristics of pooled dataset

**FIGURE 1:**
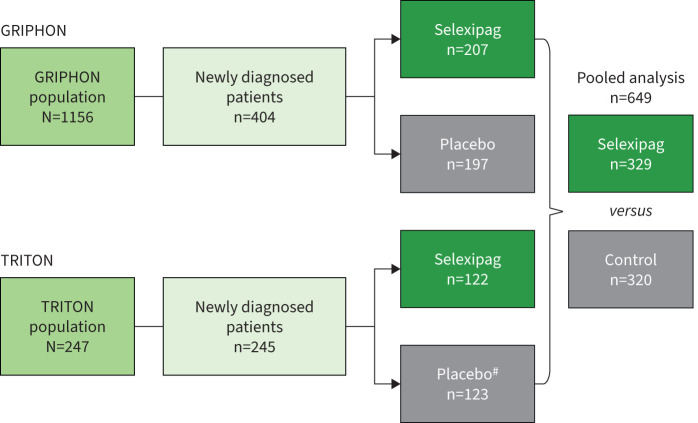
Patient disposition. Disease progression end-points defined as in GRIPHON [11] and TRITON [8], respectively, up to end of double-blind treatment +7 days. Two patients were excluded from TRITON because their time since diagnosis was over 6 months (183 days). ^#^: Placebo patients in TRITON received placebo, macitentan and tadalafil.

The combined GRIPHON and TRITON dataset included 649 patients (329 in the pooled selexipag group and 320 in the pooled control group; figure 1). Overall, the demographics and baseline characteristics of both pooled treatment groups were well balanced (table 1). In both groups, most patients (78%) were female, and most commonly had idiopathic PAH (48% in the pooled selexipag and 54% in the pooled control group). The majority of patients were in WHO FC III/IV (57% in the pooled selexipag and 62% in the pooled control group) and received selexipag/placebo as part of combination therapy (77% in the pooled selexipag group and 74% in the pooled control group). In both groups, 44% of patients were already treated with double combination therapy with an ERA+PDE-5i at baseline (table 1). Based on previous data from the individual studies, ∼47%, 30% and 21% of patients received a high (1200–1600 µg), a medium (600–1000 µg) or a low (200–400 µg) dose, respectively, of selexipag [8, 9].

**TABLE 1:** Demographics and baseline characteristics

**Characteristic**	**Pooled selexipag**	**Pooled control**
**Patients n**	329	320^#^
**Female, n (%)**	256 (77.8)	249 (77.8)
**Age years, mean±sd**	47.3±15.1	47.0±15.7
**Time since PAH diagnosis days, median (Q1–Q3)**	24.4 (12.2–82.4)	24.4 (9.2–67.1)
**PAH aetiology, n (%)**		
Idiopathic	158 (48.0)	174 (54.4)
Associated with connective tissue disease	109 (33.1)	102 (31.9)
Associated with congenital heart disease	26 (7.9)	24 (7.5)
Drug- or toxin-induced	18 (5.5)	7 (2.2)
Heritable	13 (4.0)	8 (2.5)
Associated with HIV infection	5 (1.5)	5 (1.6)
**Geographical region, n (%)**		
North America	84 (25.5)	82 (25.6)
Rest of the world	245 (74.5)	238 (74.4)
**BMI kg·m^−2^, mean±sd**	26.9±5.8	26.2±5.9
**6MWD m, mean±sd**	346.8±96.0	342.3±99.6
**NT-proBNP, n (%)** ^¶^		
Low risk (<300 ng·L^−1^)	104 (31.6)	84 (26.3)
Medium risk (300–1400 ng·L^−1^)	104 (31.6)	110 (34.4)
High risk (>1400 ng·L^−1^)	116 (35.3)	122 (38.1)
**WHO FC, n (%)**		
I/II	141 (42.9)	123 (38.4)
III/IV	188 (57.1)	197 (61.6)
**Other PAH therapy^+^, n (%)**	254 (77.2)	238 (74.4)
ERA+PDE-5i	145 (44.1)^§^	140 (43.8)
PDE-5i	88 (26.7)	77 (24.1)
ERA	21 (6.4)	21 (6.6)

PAH: pulmonary arterial hypertension; HIV: human immunodeficiency virus; BMI: body mass index; 6MWD: 6-min walk distance; NT-proBNP: N-terminal pro-brain natriuretic peptide; WHO FC: World Health Organization functional class; ERA: endothelin receptor antagonist; PDE-5i: phosphodiesterase-5 inhibitor. ^#^: n=318 for 6MWD; n=319 for BMI; ^¶^: data were missing for five selexipag and four control patients. ^+^: PAH therapy was started >3 months before randomisation, except for the 121 and 123 patients from TRITON who started ERA+PDE-5i therapy at randomisation. ^§^: one TRITON patient received background PDE-5i therapy prior to randomisation.

### Effect of selexipag on time to disease progression

In the pooled dataset, 67 (20%) patients in the pooled selexipag group experienced a first disease progression event *versus* 116 (36%) in the pooled control group (table 2). KM estimates (95% CI) for patients without an event in the pooled selexipag and pooled control groups, respectively, were 91.8% (88.0–94.4) and 84.3% (79.6–87.9) at month 6, 83.9% (79.1–87.7) and 73.0% (67.4–77.8) at month 12, and 74.3% (68.0–79.6) and 58.6% (51.7–64.9) at month 24.

**TABLE 2:** Breakdown of disease progression events

	**Pooled selexipag**	**Pooled control**
**Patients n**	329	320
**First disease progression events up to end of treatment period^#^, n (%)**	67 (20.4)	116 (36.3)
Hospitalisation for worsening of PAH	29 (8.8)	54 (16.9)
Clinical worsening of PAH^¶^	18 (5.5)	45 (14.1)
Death	14 (4.3)	13 (4.1)
Initiation of prostacyclin for worsening of PAH	6 (1.8)	4 (1.3)
**Deaths up to end of study, n (%)**	40 (12.2)	55 (17.2)

PAH: pulmonary arterial hypertension; 6MWD: 6-min walk distance; WHO FC: World Health Organization functional class. ^#^: end of treatment period was defined as end of double-blind treatment +7 days in GRIPHON and end of main observation period +7 days or end of double-blind treatment +7 days in TRITON. ^¶^: disease progression in GRIPHON [11] defined as a decrease from baseline of ≥15% in 6MWD (confirmed by a second test on a different day) accompanied by a worsening in WHO FC (for the patients in WHO FC II or III at baseline) or the need for additional PAH therapy (for the patients in WHO FC III or IV at baseline); clinical worsening in TRITON [8] defined as a post-baseline decrease in 6MWD >15% from the highest 6MWD obtained at/after screening and WHO FC III/IV (both conditions confirmed at two consecutive post-baseline visits 1–21 days apart).

**FIGURE 2:**
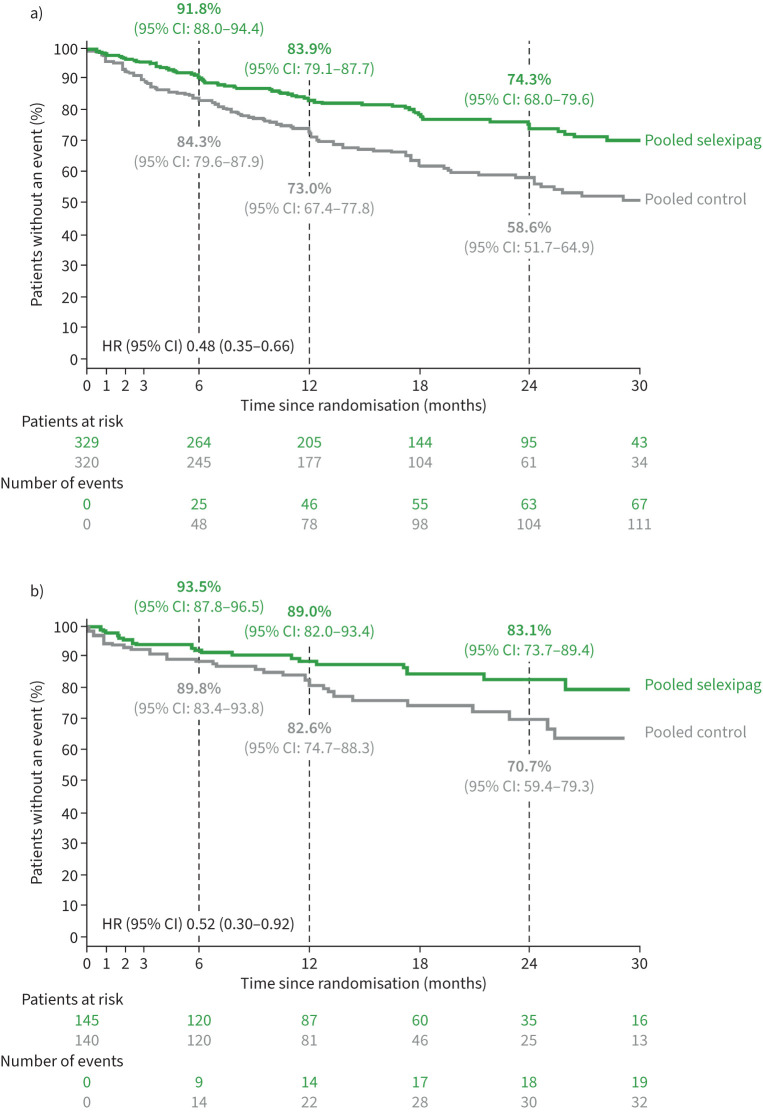
Time to disease progression up to end of treatment period in the pooled dataset: a) main analysis and b) subgroup of patients receiving ERA+PDE-5i combination therapy at randomisation. Kaplan–Meier curves illustrating time from randomisation to first disease progression event up to end of treatment period, defined as end of double-blind treatment +7 days in GRIPHON and end of main observation period +7 days or end of double-blind treatment +7 days in TRITON. Curves are cut when <10% of patients remain at risk (Pocock's rule) [13]. Kaplan–Meier estimates are shown at Months 6, 12 and 24. a) HR estimated using a Cox model which included treatment, age, sex, race, PAH aetiology, region, WHO FC, 6MWD, NT-proBNP and study as covariates. b) HR estimated using a Cox model which included treatment, region, WHO FC at baseline and study as covariates. 6MWD: 6-min walk distance; HR: hazard ratio; ERA: endothelin receptor antagonist; NT-proBNP: N-terminal pro-brain natriuretic peptide; PAH: pulmonary arterial hypertension; PDE-5i: phosphodiesterase-5 inhibitor; WHO FC: World Health Organization functional class.

Selexipag reduced the risk of disease progression by 52% (HR: 0.48; 95% CI: 0.35–0.66) compared to control (figure 2a) up to end of treatment period. This difference was driven by hospitalisation for worsening of PAH and by clinical worsening of PAH (table 2). Similar results were also obtained in the subgroup analysis of patients receiving ERA and PDE-5i double combination therapy at randomisation (n=285). In these patients, a 48% reduction in the risk of disease progression was observed for selexipag *versus* control (HR: 0.52; 95% CI: 0.30–0.92; figure 2b).

In order to assess whether other PAH-specific therapies (initiated either before or after randomisation) impacted the results, ERA and PDE-5i therapy use was added as a time-dependent variable to the same model used for the main analysis. In this analysis, the HR (95% CI) for time to first disease progression event was 0.48 (0.35–0.66) for the pooled selexipag group *versus* the pooled control group.

Matching *via* simple exact matching or propensity score weighting methods was possible for almost all patients (supplementary table S3). The treatment effect of selexipag *versus* control on disease progression for both sensitivity analyses in matched populations was consistent with the control analysis (supplementary figure S1) and the main analysis.

### Effect of selexipag on time to death

**FIGURE 3:**
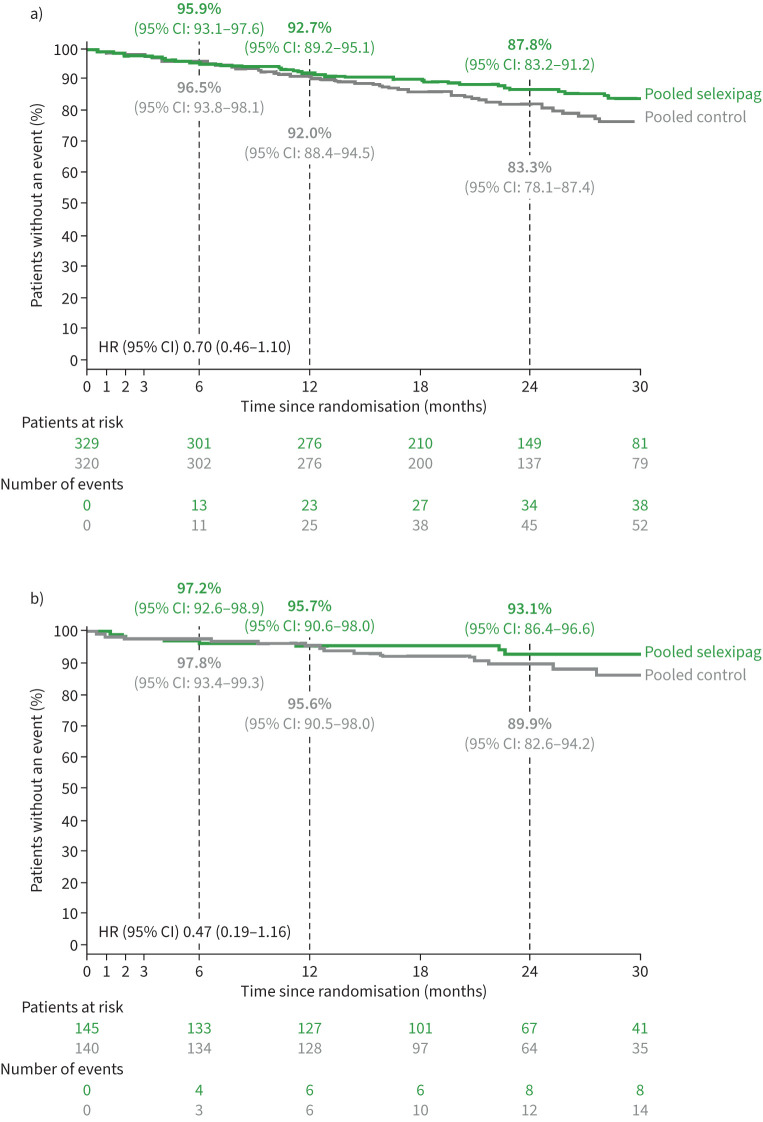
Time to all-cause death up to end of study for the pooled dataset: a) main analysis and b) subgroup of patients receiving ERA+PDE-5i therapy at randomisation. Kaplan–Meier curves illustrating time from randomisation to all-cause death up to end of study. Curves are cut when <10% of patients remain at risk (Pocock's rule) [13]. Kaplan–Meier estimates are shown at Months 6, 12 and 24. a) HR estimated using a Cox model which included treatment, age, sex, race, PAH aetiology, region, WHO FC, 6MWD, NT-proBNP and study as covariates. b) HR estimated using a Cox model which included treatment, region, WHO FC at baseline and study as covariates. 6MWD: 6-min walk distance; HR: hazard ratio; ERA: endothelin receptor antagonist; NT-proBNP: N-terminal pro-brain natriuretic peptide; PAH: pulmonary arterial hypertension; PDE-5i: phosphodiesterase-5 inhibitor; WHO FC: World Health Organization functional class.

The median (Q1–Q3) follow-up time was 25.0 (16.3–31.4) months for the pooled selexipag group and 24.2 (16.8–32.7) months for the pooled control group. The median follow-up time according to each individual study is shown in supplementary table S4. There were 40 (12.2%) deaths in the pooled selexipag group and 55 (17.2%) deaths in the pooled control group. A breakdown of deaths by individual study is shown in supplementary table S5. KM estimates (95% CI) for survival in the pooled selexipag and pooled control groups, respectively, were 95.9% (93.1–97.6) and 96.5% (93.8–98.1) at month 6, 92.7% (89.2–95.1) and 92.0% (88.4–94.5) at month 12, and 87.8% (83.2–91.2) and 83.3% (78.1–87.4) at month 24. The HR for risk of all-cause death up to end of study was 0.70 (95% CI: 0.46–1.10) for the pooled selexipag *versus* the pooled control group (figure 3a). Results were consistent in the analysis that included ERA and PDE-5i therapy use as a time-dependent covariate (HR: 0.70; 95% CI: 0.46–1.08) and in the subgroup analysis of patients receiving background therapy with an ERA and PDE-5i (HR: 0.47; 95% CI: 0.19–1.16; figure 3b).

### Safety

The median (min, max) exposure to study treatment was 16.7 (0.1, 42.0) and 13.4 (0.1, 43.2) months in the pooled selexipag and pooled control groups, respectively (table 3). The most common AEs are shown in table 3, with the most frequent being headache, diarrhoea, nausea and peripheral oedema. There were 85 (26%) patients in the pooled selexipag group and 100 (31%) in the pooled control group who discontinued treatment due to an AE (table 3).

**TABLE 3:** Exposure and safety

	**Pooled selexipag**	**Pooled control**
**Patients n**	329	320
**Exposure to study treatment months, median (min, max)**	16.7 (0.1, 42.0)	13.4 (0.1, 43.2)
**AEs, n (%)**		
Patients with ≥1 AE	323 (98.2)	307 (95.9)
Patients with ≥1 serious AE	140 (42.6)	129 (40.3)
Patients with ≥1 AE leading to discontinuation of double-blind study treatment	85 (25.8)	100 (31.3)
**Number of AEs**	2836	2259
**Most frequent AEs, n (%)^#^**		
Headache	208 (7.3)	126 (5.5)
Diarrhoea	130 (4.6)	64 (2.8)
Nausea	105 (3.7)	51 (2.2)
Peripheral oedema	79 (2.8)	76 (3.3)
Pain in jaw	68 (2.4)	20 (0.9)
Pain in extremity	65 (2.3)	24 (1.1)
Vomiting	62 (2.2)	29 (1.3)
PAH worsening	53 (1.9)	94 (4.1)
Dyspnoea	49 (1.7)	61 (2.7)
Myalgia	48 (1.7)	33 (1.5)
Arthralgia	44 (1.5)	29 (1.3)
Dizziness	41 (1.4)	47 (2.1)
Nasopharyngitis	40 (1.4)	34 (1.5)
Flushing	38 (1.3)	26 (1.1)
Cough	34 (1.2)	37 (1.6)
Upper respiratory tract infection	33 (1.2)	46 (2.0)
Fatigue	32 (1.1)	33 (1.5)
Dyspepsia	32 (1.1)	18 (0.8)
Anaemia	31 (1.1)	21 (0.9)
Back pain	23 (0.8)	24 (1.1)
Nasal congestion	22 (0.8)	24 (1.1)
Right ventricular failure	18 (0.6)	26 (1.1)
Gastroesophageal reflux disease	16 (0.6)	22 (1.0)

AE: adverse event; PAH: pulmonary arterial hypertension. ^#^: calculated based on the number of AEs; includes AEs with a frequency of ≥1% in either group.

## Discussion

This *post hoc* analysis of pooled data from the GRIPHON and TRITON clinical trials showed that selexipag treatment initiated within 6 months of diagnosis reduced the risk of disease progression in a large population of PAH patients that included >600 patients, a rarity in PAH analyses, with many receiving double background therapy with an ERA and PDE-5i. Selexipag halved the risk of disease progression, and a consistent treatment effect was observed when selexipag was administered within 6 months of diagnosis as part of a triple therapy regimen. Similar to other studies in PAH that assess disease progression, this treatment effect was driven by PAH worsening and PAH-related hospitalisation [8, 11, 17, 18]. The nature and severity of the reported AEs reflect the safety profile of selexipag and the underlying morbidity and/or mortality of PAH populations.

PAH is a progressive disease with a current treatment paradigm recommending multiple therapies, often in combination [3–5]. In this context, it is important to consider the long-term management of patients with the goal to delay disease progression. In the GRIPHON study, selexipag significantly delayed disease progression, especially in newly diagnosed patients, and led to a moderate effect on 6MWD [9, 11]. In the TRITON study, there was no effect of selexipag compared to placebo on short-term end-points, such as haemodynamic status or 6MWD, but there was a signal that early selexipag treatment may reduce the risk of PAH disease progression. The current analysis complements this latter finding by demonstrating consistent results in a larger dataset with assessment of a greater number of events. In addition, the pooled survival data show a trend consistent with the data for time to disease progression. Previous data have indicated that the treatment effect of selexipag on disease progression is not impacted by use of other PAH therapies [9, 11] which is also demonstrated by the results reported here. Accounting for the potential impact of other PAH therapies did not affect the benefit of early selexipag initiation on disease progression observed in the pooled cohort, including in patients for whom selexipag was added on top of double oral combination therapy (*i.e.*, ERA and PDE-5i combination therapy). These data support the signal observed in TRITON for a reduced risk of disease progression with initial triple *versus* initial double combination therapy. However, as short-term haemodynamic or functional parameters such as 6MWD were not investigated within this analysis, no additional insights could be gained into the discrepancy between the effect of triple *versus* double oral combination therapy on short-term haemodynamic and functional parameters *versus* long-term outcomes. These results are also important as they support the targeting of the three established pathogenic pathways in PAH [1] within 6 months of diagnosis and are in line with the recommendations for combination therapy in today's management of PAH patients [3–5]. However, as data were only available on the treatment effect of selexipag added to an ERA+PDE-5i, no conclusions could be drawn on what the outcomes on disease progression might have been with different treatment combinations.

Pooling data from GRIPHON and TRITON was considered appropriate as time to disease progression was assessed in a similar way in both studies, albeit as a primary end-point in GRIPHON and as a secondary end-point (in an exploratory analysis) in TRITON. These end-points were assessed over a comparable follow-up time and with similar censoring rules applied [8, 11]. Both studies allowed long-term analysis of time to disease progression and were similar in design and included similar study populations of newly diagnosed patients, further supporting the appropriateness to pool data from these two studies. Consistent results were also observed in additional analyses using time-dependent variables and sensitivity analyses that used patient matching methodologies to account for other differences between groups. Pooling data from clinical trials, taking into account the associated caveats, allows analyses in larger populations [19], which is specifically relevant in rare diseases, such as PAH. Here, it provided the opportunity to further investigate the efficacy of early initiation of selexipag on outcomes in a large population of >600 PAH patients.

This *post hoc* analysis of pooled clinical trial data is subject to limitations. No systematic review to identify additional studies in databases or registries was performed, and therefore results were not reported according to the PRISMA guidelines [20]. Pooling of the data from both clinical trials was performed at the patient level. For the analysis of the subgroup of patients receiving double combination therapy at randomisation fewer variables were included in the model as compared to that of the main model due to patient and event numbers. The confidence intervals for analyses in this subgroup were wide, especially for the time to all-cause death analysis, and the results should be interpreted with caution. Despite the strong similarities between the two studies, and although sensitivity analyses were performed to show that results were consistent across studies, some differences may still have impacted the current results. These include possible variations in clinical practice at the time of the studies (enrolment for GRIPHON was between 2009 and 2013 whereas screening for TRITON was between 2016 and 2018) and differences in the countries/sites present (GRIPHON was conducted worldwide while TRITON was conducted in North America and Europe).

### Conclusions

This *post hoc* analysis of pooled data from the GRIPHON and TRITON clinical trials suggests that selexipag use within 6 months of diagnosis is beneficial in delaying disease progression in PAH, including as part of a combination therapy regimen.

## Supplementary material

10.1183/23120541.00456-2022.Supp1 **Please note:** supplementary material is not edited by the Editorial Office, and is uploaded as it has been supplied by the author.
Supplementary material 00456-2022.SUPPLEMENT
