# Supplementary material for: Early selexipag initiation and long-term outcomes: insights from randomised controlled trials in pulmonary arterial hypertension
Source: ERJ Open Res. 2023 Jan 16;9(1):00456-2022. doi: 10.1183/23120541.00456-2022 (PMC9841313; doi:10.1183/23120541.00456-2022)
Supplement: Supplementary file 1 [file 00456-2022.SUPPLEMENT.pdf]

Supplementary Appendix

	**Page**
Supplementary Methods	2
**Table S1.** Overview of GRIPHON and TRITON study designs	3
**Table S2.** Components of endpoints in GRIPHON and TRITON	5
**Table S3.** Overview of patients included in analysis sets	6
**Table S4.** Median follow-up time	7
**Table S5.** Summary of deaths at the end of the analysis period	8
**Figure S1.** Time to disease progression up to end of treatment period for (A) the pooled analysis set (B) the pooled matched analysis set and (C) the pooled weighted analysis set	9

Supplementary Methods

1. Sensitivity analyses

- In order to explore potential differences and confounding factors between the pooled control and pooled selexipag groups in the pooled dataset, simple exact matching and propensity score weighting analyses were applied.
    - Pooled matched analysis set: Simple exact matching included matching a participant in the pooled selexipag group to a participant in the pooled control group on the baseline variables WHO FC and region.
    - Pooled weighted analysis set: Propensity score weighting matching analysis used the baseline characteristics age, sex, race, PAH aetiology, region, WHO functional class (FC), 6MWD, N-terminal pro-brain natriuretic peptide (NT-proBNP) levels and study to make the distribution of observed baseline characteristics similar between pooled control and pooled selexipag groups.
        - A logistic model that includes these baseline characteristics was used to estimate the propensity score.
        - The average treatment effect overlap was applied and the standardised mean differences were calculated to check the balance of covariates.
    - Time to disease progression was then analysed using a similar Cox proportional hazard model as for the main analysis. The control analysis based on the full analysis set used treatment, region, WHO FC at baseline, and study as covariates; the analyses using the pooled matched and pooled weighted analysis sets used treatment only as a covariate.

Table S1. Overview of GRIPHON and TRITON study designs

Study	GRIPHON[11]	TRITON[8]
**Summary**	Double-blind, randomised, placebo-controlled, event-driven phase III study.	Double-blind, randomised, placebo-controlled phase IIIb study.
**Patients**	1156 patients were randomised:<br>• 574 received selexipag and 582 received placebo twice daily.	247 patients were randomised:<br>• 123 patients received initial triple oral therapy (macitentan, tadalafil, and selexipag) and 124 patients received initial double oral therapy (macitentan, tadalafil, and placebo).
**Study treatments**	• Study drug was titrated over a 12-week period to reach an individualised maintenance dose (ranging from 200 to 1600 µg twice daily).<br>• Patients received double-blind treatment until either they experienced a primary endpoint event, they prematurely discontinued study drug, or the end of the study was declared.	• Open-label macitentan 10 mg once daily and tadalafil 20 mg once daily were initiated on Day 1. Tadalafil was increased to 40 mg once daily on Day 8 ± 3 according to tolerability.<br>• Double-blind selexipag or placebo were initiated on Day 15 ± 3 at 200 µg twice daily, then titrated up to Week 12 to reach an individualised maintenance dose (ranging from 200 to 1600 µg twice daily).<br>• Study treatments were administered until the last randomised patient reached Week 26 (end of the main observation period).
**Selection Criteria**	• PAH patients (18-75 years of age) diagnosed by right heart catheterisation with a PVR ≥5 WU.<br>• Patients had a 6MWD of 50 to	• PAH patients (18-75 years of age) diagnosed by right heart catheterisation within 6 months prior to randomisation with a PVR ≥6 WU.

	450 m at screening.<br>• Patients were treatment-naïve or receiving a PDE5-i, ERA, or both at stable doses for ≤3 months prior to randomisation.	• Patients had a 6MWD ≥50 m<br>• Patients were excluded if previously treated with PAH therapy.

6MWD: 6-minute walk distance; ERA: endothelin receptor antagonist; PAH: pulmonary arterial hypertension; PDE5-i: phosphodiesterase type 5 inhibitor; PVR: pulmonary vascular resistance; WU: Wood Units.

Table S2. Components of endpoints in GRIPHON and TRITON

**Study**	**GRIPHON[11]**	**TRITON[8]**
**Endpoint**	Morbidity/mortality (primary endpoint)	Time to disease progression (secondary endpoint)
**Components**	All-cause death	All-cause death
	Hospitalisation for worsening PAH	Hospitalisation for worsening PAH
	Initiation of parenteral prostanoid therapy or long-term oxygen therapy for worsening PAH	Initiation of prostacyclin, a prostacyclin analog, or prostacyclin receptor agonist for worsening PAH
	Disease progression, defined as:<br>• A decrease from baseline of at least 15% in the 6MWD (confirmed by means of a second test on a different day) accompanied by a worsening in WHO FC (for the patients with WHO FC II or III at baseline) or<br>• the need for additional treatment of PAH (for the patients with WHO FC III or IV at baseline)	Clinical worsening defined as:<br>• A post-baseline decrease in 6MWD > 15% from the highest 6MWD obtained at/after screening and<br>• WHO FC III/IV (both conditions confirmed at two consecutive post-baseline visits 1–21 days apart)
	The need for lung transplantation or balloon atrial septostomy on account of worsening PAH, as judged by the physician	

6MWD: 6-minute walk distance; PAH: pulmonary arterial hypertension; WHO FC: World Health Organization functional class.

Table S3. Overview of patients included in analysis sets

	**Pooled Selexipag**<br><br>**N = 329**	**Pooled Control**<br><br>**N = 320**	**Total**<br><br>**N = 649**
Main analysis: Pooled dataset, n (%)	329 (100)	320 (100)	649 **(100)**
Sensitivity analyses, n (%):			
Pooled matched analysis set	307 (93.3)	307 (95.9)	614 (**94.6)**
Pooled weighted analysis set	319 (97.0)	309 (96.6)	628 **(96.8)**

Pooled matched analysis set: Each patient in the pooled selexipag group was matched to a patient in the pooled control group based on region and baseline WHO FC. Patients who could not be matched were excluded (pooled selexipag: 22; pooled control: 13). Pooled weighted analysis set: Propensity scores were estimated from logistic regression based on baseline covariates (age, sex, race, PAH aetiology, region, WHO FC, 6-minute walk distance, NT-proBNP, and study). Patients who had missing values preventing the calculation of the propensity score were excluded (pooled selexipag: 10; pooled control: 13). PAH: pulmonary arterial hypertension; NT-proBNP: N-terminal pro-brain natriuretic peptide; WHO FC: World Health Organization functional class.

Table S4. Median follow-up time

	Pooled analysis		GRIPHON		TRITON	
	**Pooled Selexipag N = 329**	**Pooled Control N = 320**	**Selexipag N = 207**	**Placebo N = 197**	**Initial triple therapy N = 122**	**Initial double therapy N = 123**
Follow-up time, months, median (Q1, Q3)	25.0 (16.3, 31.4)	24.2 (16.8, 32.7)	25.6 (16.1, 30.6)	24.9 (16.0, 33.1)	24.1 (17.1, 32.4)	23.4 (18.2, 32.1)

Table S5. Summary of deaths at the end of the analysis period

	**Pooled Selexipag**<br>**N = 329**	**Pooled Control**<br>**N = 320**
Patients who died, n (%)		
GRIPHON and TRITON	40 (12.2)	55 (17.2)
GRIPHON	36 (10.9)	43 (13.4)
TRITON	4 (1.2)	12 (3.8)

Figure S1. Time to disease progression up to end of treatment period for (A) the pooled analysis set (B) the pooled matched analysis set and (C) the pooled weighted analysis set

A)

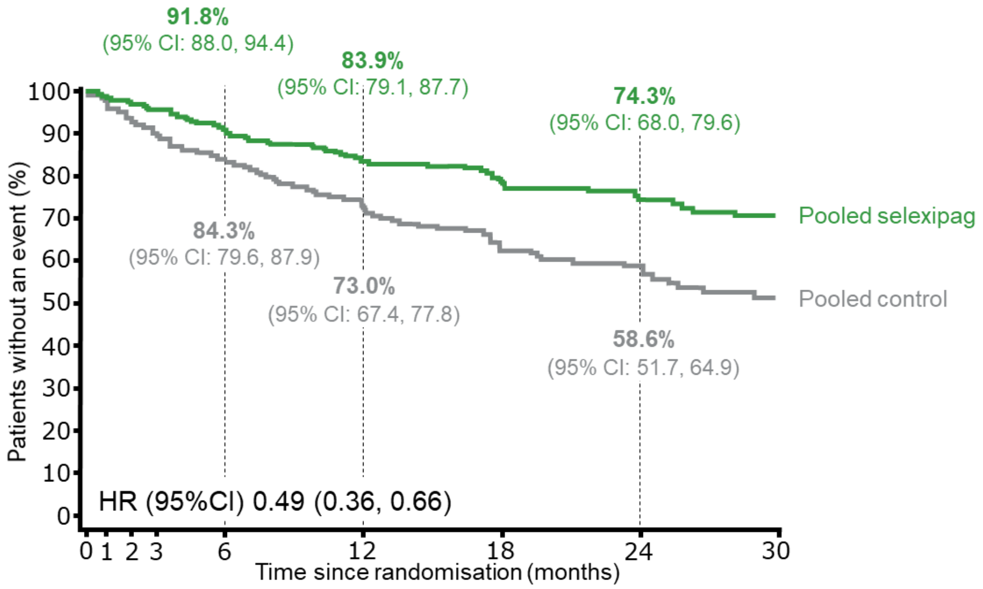

Patients at risk

329	264	205	144	95	43
320	245	177	104	61	34

Number of events

0	25	46	55	63	67
0	48	78	98	104	111

B)

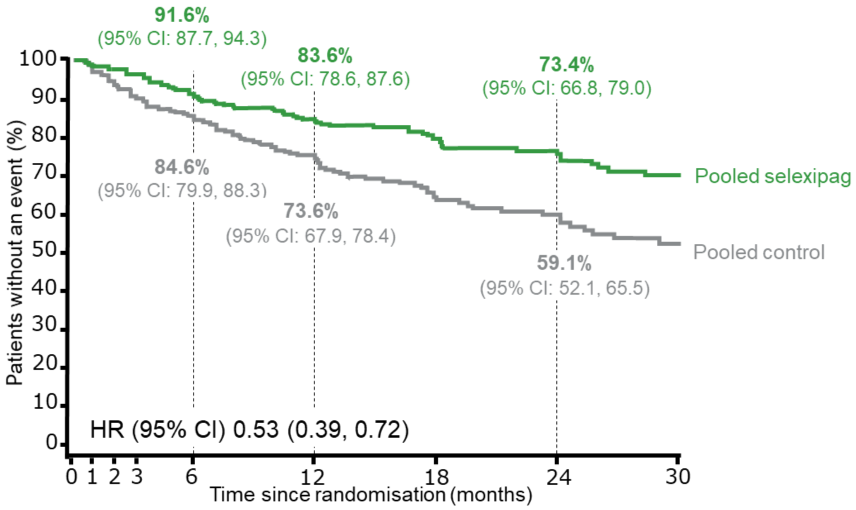

Patients at risk

307	247	192	134	89	40
307	236	170	100	58	33

Number of events

0	24	44	53	61	65
0	45	73	92	98	105

C)

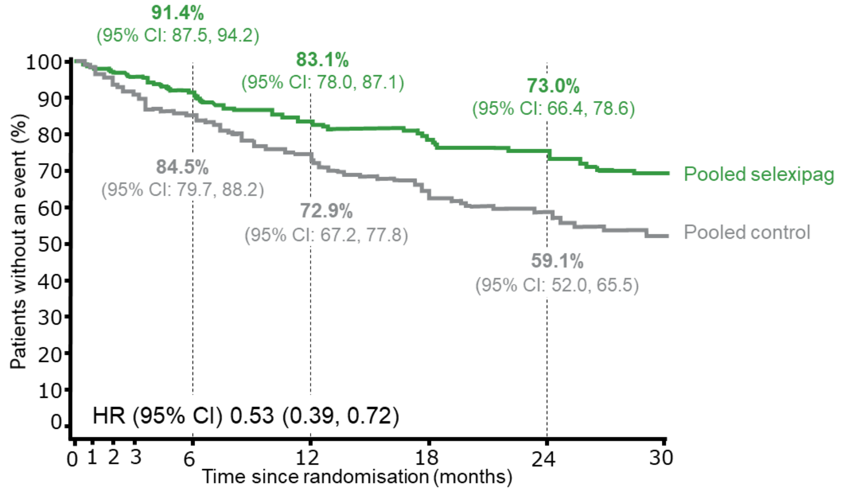

Patients at risk						
	319	255.5	195.6	137.1	91.4	41.8
	309	237.9	170.9	100.9	58.5	33.4
Number of events						
	0	25.2	46.9	55.7	63.9	68.0
	0	45.7	75.6	93.9	99.5	105.5

Kaplan-Meier curves illustrating time from randomisation to first disease progression event up to end of treatment period, defined as end of double-blind treatment + 7 days in GRIPHON and end of main observation period + 7 days or end of double-blind treatment + 7 days in TRITON. Curves are cut when < 10% of patients remain at risk. Kaplan-Meier estimates are shown at Months 6, 12 and 24. A) HR estimated using a Cox model which included treatment, region, WHO FC at baseline, and study as covariates. B) HR estimated using a Cox model which included treatment as a covariate. C) HR estimated using a Cox model which included treatment as a covariate and age, sex, race, PAH aetiology, region, WHO FC, 6MWD, NT-proBNP, and study as variables for the propensity score. 6MWD: 6-minute walk distance; HR: hazard ratio; ERA: endothelin receptor antagonist; NT-proBNP: N-terminal pro-brain natriuretic peptide; PAH: pulmonary arterial hypertension; PDE-5i: phosphodiesterase-5 inhibitor; WHO FC: World Health Organization functional class.
